# A Narrative Evaluation of a Grief Support Camp for Families Affected by a Parent's Suicide

**DOI:** 10.3389/fpsyt.2021.783066

**Published:** 2021-11-30

**Authors:** Anneli Silvén Hagström

**Affiliations:** Department of Social Work, Stockholm University, Stockholm, Sweden

**Keywords:** bereavement, children, family intervention, grief support, mental health, narrative evaluation, stigmatization, suicide

## Abstract

**Background:** Children of parents who suffer mental ill-health and die by suicide are vulnerable to developing psychological and social problems themselves; they also have a severely elevated risk of dying at a young age – particularly through suicide. This highlights the need to design supportive measures that can counteract such negative developments after a parent's suicide.

**Aim:** This narrative evaluation of a grief support camp for families affected by a parent's suicide arranged by the non-profit organization Children's Rights in Society in Sweden investigates *whether* children [*N* = 11] and parents [*N* = 11] perceived their participation as meaningful and, if so, *in what way*, and the *changes* to which the program was said to have contributed.

**Methods:** Family members were invited to reflect on their experiences in narratively structured interviews that took place 18 months after participation. Their narrated experiences were analyzed to examine how the program was integrated into their biographies and with what significance. Narratives of change were identified in particular in order to grasp the self-perceived effects of participation.

**Results:** Both children and parents attributed major significance to their encounters with other suicide bereaved. This led to support exchange and normalization, which countered a perceived “suicide stigma” in everyday life. Help to narratively construct destigmatizing understandings of suicide was also said to have relieved self-blame and shame. Overall, the participants described changes in the form of a better-informed position in grief, increased manageability and enhanced family communication. The parents also reported improved ability to support their children and a more hopeful view of life ahead.

**Conclusion:** The evaluation showcases how this psychoeducational intervention, at a relatively low cost compared to traditional approaches, has great potential to lessen the negative effects of a suicide in the family by assisting families with psychological processing and de-stigmatization. Parental resources are also strengthened, which can serve as continuing support for the children.

## Rationale

The Swedish Health and Medical Services Act (SFS 2017:30, chap 5: 7§) emphasizes the responsibility of health and medical care to provide information, advice and support to a child if her or his parent, or another adult with whom the child lives permanently, suffers from a mental illness or disability, and/or unexpectedly dies (1). However, children who have lost a parent through suicide are a neglected group in Swedish society. In addition, the United Nations Convention on the Rights of the Child has been binding Swedish law since January 2020. This further accentuates the right of parentally suicide-bereaved children to access to age-appropriate information and support. However, neither the national guidelines nor legislation stipulate *the kind of professional support* that should be provided to children who suffer the loss of a parent through suicide. Moreover, although stipulated as mandatory, in practice professional support is only offered exceptionally to such children and their families. One consequence of this failure to routinely offer support is that the remaining parent must be attentive to the child's processing of loss and active in the search for professional support where deemed necessary. This can be a difficult task, however, as parents must manage the effects of their own grief and mourning children may hide their grief to avoid worrying or burdening the grieving parent. In addition, children's access to professional assistance with grief is fully dependent on the local availability of professional bereavement counseling and peer-support groups. These circumstances mean that a considerable number of children must cope without professional grief support after a parent's suicide, due to the remaining parent's lack of initiative or know-how regarding *whether* and *where* to seek professional support, and/or a general shortage of professional grief interventions—especially those which specialize in suicide-bereavement. At the same time, previous studies in the field have established that children who have lost a parent through suicide constitute a risk group for developing complicated grief due to the difficulties of resolving the loss. This has been linked to psychiatric morbidity (e.g., anxiety, depression, PTSD, suicidal ideation) as well as social problems (2). As a result, suicide-bereaved children have a severely elevated risk of dying at a young age—particularly through suicide (3, 4). There is thus a critical need to provide these children with appropriate post-vention measures that can cater for their specific needs in grief and prevent such an accumulation of adverse effects after a parent's suicide.

Since 2013, Children's Rights in Society (Barnens rätt i samhället, BRIS) has organized a grief support program in the format of a weekend camp—known as support weekends—for families affected by a parent's suicide. The primary objectives of this psychoeducational program are to help children and their parents to: (a) identify how life has changed since the suicide loss, and their own responses and needs in grief; (b) develop health-promoting coping strategies; and (c) facilitate open and supportive family communication. A further main aim is to assist parents through dialogue to develop their skills to understand and support their children in grief. This is an exception to the otherwise absence of grief support programs directed at suicide-bereaved children and their families in Sweden.

Knowledge of the perceived meaningfulness and takeaways from similar grief support programs is scant and, to date, few studies have analyzed suicide-bereaved children's and parents' responses to their participation in such programs. This might be explained by the fact that grief support programs that specialize in suicide bereavement are still rare in many countries, and that existing programs have been evaluated first and foremost using quantitative methods, such as questionnaires, that provide pre-printed response options. The few existing studies have importantly concluded that family-based interventions for suicide-bereaved children can lessen suicide-related distress and promote children's emotional and social functioning in grief (5, 6). However, less is known about how these effects are achieved and how the program content has been integrated into suicide bereaved families' grief processes and lives. Hence, children and their parents have only to a limited extent been encouraged to talk freely about their experiences, and to consider how the program was located within their ongoing biographies and with what significance. This is the distinct purpose of this article, in which children's and parents' narrated experiences of their participation in the BRIS grief support program for families affected by a parent's suicide are analyzed to investigate *whether* they perceived the program to be meaningful and, if so, *in what ways*. Of particular interest is to identify so-called *narratives of change* in order to capture how the grief support program is said to have contributed to actual changes in the participants' grieving processes and lives.

## Parental Suicide-Bereaved Children's Grief Experiences and Needs

Although many children experience the fatal loss of a loved one in childhood, their grief tends to be overlooked by adults who commonly avoid talking with children about death and the deceased, which contributes to a powerlessness in young people's dealing with loss (7). This is particularly evident in cases of unnatural deaths, such as through suicide (8). Following a parent's suicide, in addition to the child's age and maturity/ability to conceptualize death, the supportive role of the remaining parent and an open communication climate in the family have been identified as vital to the ability of children to cope with the loss (9). However, distorted communication commonly occurs due to the remaining parent's efforts to protect the child from the circumstances of the suicide (10). Such concealment may, contrary to its aim, complicate the child's processing of loss and leave the child in a confused and lonely position in grief.

Parentally suicide-bereaved children are also faced with a “double whammy”; in addition to coping with the loss itself, children are left to try to make sense of their parent's suicide [(8), p. 192]. The question, “*Why* did my mother/father *choose* to die?,” is central to young mourners' meaning reconstruction after a parent's suicide (11, 12). The search for answers together with the lack of information from adults commonly produce self-blame and shame, as parentally suicide-bereaved children^1^ for various reasons tend to take the blame for the suicide on themselves. They may also hold the deceased parent accountable, based on the belief that the parent failed in his or her moral responsibility to care for them, and for selfish reasons chose to leave the child. Both understandings produce a stigmatized identity influenced by anger, shame and blame, either as a “failed” daughter/son or as the offspring of a deeply “immoral person” (12). At the heart of suicide-bereaved children's stigmatization is the sense of having been unloved and/or abandoned by the deceased parent, which ultimately raises questions about the child's self-worth [ibid.; (8)]. This culturally induced “suicide stigma” can be reinforced by non-supportive responses within the children's social network, such as straightforward questions from other children, “is there crazy in your family?” [(8), p. 192], or avoidance and outright rejection (12). Stigmatization has been shown to play a central role in suicide bereavement (13) and in research parentally suicide-bereaved children describe themselves as feeling deviant—and even strange or tainted—by their parent's suicide (14, 15).

Where a parental suicide-bereaved child's and the remaining parent's need for support in grief is substantial and the social support is inadequate, access to professional interventions becomes critical. However, children and families are seldom offered professional support in connection with a family member's suicide (16). Young mourners may also be dissatisfied with the professional support they receive, due to a perceived lack of empathy and knowledge about grief after suicide among professionals (17).

## Evaluation Studies in the Field

A systematic review of the effects of grief support programs for parentally bereaved children shows that when the remaining parent is supported, there is an improvement in parental health in grief and an enhanced capacity to care for the child, leading to positive effects on children's grieving (18). Another advantage of a family-based approach is the opportunity for children and parents to sit down and talk about parental loss together—sometimes for the first time. Grief interventions for a parentally bereaved child together with the remaining parent are therefore generally recommended.

A study examining children's experiences of participation in grief support programs shows that a combination of camp-specific activities and therapeutic conversations/exercises contributed to an improvement in the children's perceived well-being (19). Participation in activities is said to contribute to community, belonging and strengthened self-esteem, and to offer a break from painful emotions. The therapeutic conversations and exercises, in turn, are said to contribute to healing in grief, to understanding and putting words to experiences, to assisting with memory and to forming a continuing bond with the deceased. A review of the effectiveness of bereavement camps for children (20) confirms that this combination of a safe space to express grief in a therapeutic environment in the company of other bereaved children and playful activities is a promising venue to help bereaved children to build resilience.

Nonetheless, the effectiveness of general grief interventions with suicide-bereaved children has been questioned due to weak results (21). It has even been argued by support-group practitioners that suicide-bereaved children may experience reinforced stigmatization in these blended contexts, where they maintain silence about the circumstances of the death (8). Instead, specialized programs led by trained facilitators that take aspects such as the social environment into account yield more promising results (22, 23). An evaluation of a grief support program aimed at suicide-bereaved children and their parents (6), focused on children's reactions to death and suicide, and on strengthening their coping skills, demonstrated a significant reduction in anxiety and depressive symptoms in children. The evaluation of another family-based program focused on children's emotional needs (5) showed a similar reduction in anxiety and depressive symptoms, as well of disruptive behavior in bereaved children. The program also increased knowledge, self-esteem and agency, and led to more successful coping. Although research on suicide-specific support programs for both parentally suicide-bereaved children and their remaining parents is still scant, there are weak but promising indications that family-based programs can help to improve children's coping with parental loss and reduce suicide-specific symptoms of grief connected to complicated grief. There is, however, an urgent need to examine how suicide-bereaved families themselves experience such programs and what they find helpful.

## Materials and Methods

### A Narrative Approach to Program Evaluation

Quantitative methods dominate the evaluation field and a narrative approach to program evaluation is much rarer. Narrative inquiry investigates how people make sense of events, the world they live in and their related identities. Hence, the stories people tell reveal subjective truths about their lives and identities, and offer context-specific knowledge that might not always be discovered using other methods. A main focus of narrative program evaluation is *change*. Baú (24) encourages the researcher to ask people to recognize change when recounting their biography from past to present, including the professional intervention, as this makes it possible to understand how people integrate the program content and how it is applied in their continued living. A narrative approach entails the notion that evaluation is not the endpoint of applied knowledge but a contributor of new culture-specific knowledge. Such knowledge production also comes in the form of a narrative. According to Constantino and Greene [(25), p. 47]: “By telling the program's story, an evaluation may be used to give voice to participants' perspectives, as they and their experiences provide the characters and events of the program's narrative.” Like an evaluation story, this article constructs an overall meaning of the participants' experiences and takeaways from the program, with the aim of examining the difference made by the program from a wider social and cultural perspective.

### Theoretical Basis, Structure and Thematic of the Grief Support Program

The BRIS grief support program for families affected by a parent's suicide comprises two support weekends, Friday to Sunday, 4 months apart. On each occasion 10–12 families participate. This nationwide program is subsidized by the public health authority and located in the middle of Sweden, enabling families from different socio-economic backgrounds and localities to participate. Program information is published on the BRIS website and on social media, and is also distributed to suicide bereavement and mental health organizations. Although the program has a family-based approach, its main objective is to facilitate children's grief. The program is based on a systems theory perspective and the notion that family members' grief is interrelated [see (26, 27)]. Families' post-loss communication and interaction are thus understood as essential to suicide-bereaved children's abilities to cope with grief. The salutogenic perspective ‘Sense of coherence’ (28) also has a central role in the program. It is based on the notion that honest and age-appropriate information, space and support for expression and reflection, as well as help to develop resilient coping strategies can strengthen children's comprehension and the manageability of parental loss, and contribute to increased meaningfulness in life. The program also draws on theories about childhood grief from an attachment and development perspective (29).

The program is structured around parallel group meetings, where parents and children in parallel but separately process the same themes adapted to age. The composition of the children's groups is based on the current participants and divided according to age. The children in the youngest age group are 4–6 years old and the oldest children are 20 years old. Each group consists of 4–8 children. All the parents are in one group. The main themes processed in the groups are: “Information about suicide and suicide bereavement”; “The family then and now: what happened?”; “What has changed?”; “Grief responses and emotions”; “My grief/others' grief”; “Remembering the deceased parent”; “What helps and how do I take care of myself?”; “Questions I wanted to ask but have not dared”; and “What is my future?” (26). These sessions are combined with grief-oriented family exercises and playful activities, where the latter offer opportunities for relaxation and togetherness within families and between participants (for a fuller description of the program content, see Supplementary Material). The psychoeducational components of the program focus on helping the children to express their thoughts and feelings about their parental loss. Participants also learn about why people die by suicide, common grief responses and needs, and strategies for coping with grief, viewed over time [i.e., the oscillation between loss-oriented and restoration-oriented coping, (27)]. In addition, the children are supported to construct a narrative about their deceased parent and identify positive memories and parental attributes with which the child may identify. Psychoeducational components support the parents to understand childhood bereavement, foster their children's expression and emotional and social functioning in grief, and open up space for family conversations about the deceased parent and grief. The parents also ventilate their own grief, but with primary attention on their parenting role.

### Study Design and Procedure

The author is a social worker, grief therapist and researcher who specializes in young people's grief after a parent's suicide. She has long clinical experience of talking with children and teenagers about sensitive issues related to family problems and loss, which was gained in child and adolescent psychiatric care. She was asked to conduct an evaluation of the current program without having had any pre-existing relationship with the organizer or any of the personnel. The study was carried out in three steps. First, the author conducted participant observations at the grief support camp on two separate occasions to understand the context and program content, and to observe the knowledge and support exchange through exercises and activities, as well as the social interaction between the professionals (social workers specialized in children's grief) and participating family members—and between the participants themselves. An additional aim was to make contact with the participants, primarily the children and teenagers, in order to build trust, which should have a positive effect on participation in the interviews. Second, all the participants were informed about the study orally by the author and in writing on the first program day, and later invited to take part in the study in an age-appropriate and personally addressed letter followed-up by a telephone contact with the parent of each family. Third, all those who agreed to contribute were interviewed 18 months after the program ended. Two children decided to participate only after meeting the author in connection with interviews with other family members.

### Study Participants and Material

All the members of the 14 families that attended the BRIS grief support camp on two different occasions between 2017 and 2018 were invited to participate in the study [*N* = 49]. Of these, 11 children (six girls and five boys), aged between six and 13 with a mean age of nine at the time of their participation, and 11 parents (nine mothers and two fathers) [*N* = 22] from eight families agreed to be interviewed for the study. The time elapsed from the parental loss to program participation varied between 6 months and 5 years, with an average of about 1.5 years. There were variations in urban and rural locations, and socio-economic and cultural backgrounds but ethnic Swedish, middle class families were predominant. The interviews were conducted in-person in the families' homes, and with children and parents separately. A general feature of the interviews, which were adapted according to the children's age and maturity, was that the participants were asked to talk about what life was like before and after participation, and how the program content was perceived and thought to have contributed to grieving and life in general. Special attention was therefore paid to descriptions of daily life, grief reactions, coping strategies and support needs. The material differed in narrative richness and the younger children in particular needed to be more actively supported and reminded of various activities before they could engage in storytelling. The interviews were recorded and transcribed verbatim for analysis. The participant observations mainly contributed to the author being better informed during the interviews but were also used to contextualize the study results.

### Analysis of the Participants' Narrated Experiences

The analysis was guided by a narrative methodology for evaluation to investigate *whether* the participating parents and children perceived the program to be meaningful and, if so, *in what ways*. Of particular interest was to identify so-called *narratives of change* in order to capture how the grief support program is said to have contributed to actual changes in the participants' grieving processes and lives. The participants' narrated experiences constructed in research interviews were analyzed using narrative methodology (30), and the concept of “narratives of change” (24). First, the transcriptions were read repeatedly to identify the narrative thematic of the meanings attributed to participation in the BRIS grief support camp. Narratives of change were then delineated and thematically analyzed to grasp the perceived impact of the program on the participants' ongoing grieving processes and lives. The children's and parents' narratives were first analyzed separately and later compared to construct a more complex understanding of each family situation, and find connections and differences in the material. The results have been discussed and validated against the interview material at a research seminar with narrative researchers.

### Ethical Considerations

Interviewing children about potentially traumatic and stigmatizing experiences such as the death of a parent through suicide is an ethically sensitive issue. It can stir up unresolved issues and emotions linked to the loss and actualize a need for professional support. The interview situation itself, between an adult interviewer and a child, is also unequal and constitutes an imbalance of power that can incline children to adapt to what they believe is expected of them and ignore their own needs (31). Based on this, the research interviews were conducted with great sensitivity and respect for each child's integrity and personal needs; for example, two sisters chose to be interviewed together and many children chose to make drawings during the interview. The narrative approach facilitated the children to decide for themselves what they wanted to disclose. They were instructed to tell only what they wanted to tell, no matter how much or how little, and to just say “I do not want to talk about it” if they did not want to answer a question by the author. The children usually recognized the author and in conversations before the interview the author discussed memories of the camp to establish contact. Another facilitator for the children to express themselves was that they had all participated in a support group activity and thus to some extent acquired a language for talking about their suicide loss experience. At the end of each interview, the children were asked how they felt after having talked about their loss and grief experiences. Although the interviews brought up painful thoughts and emotions, all the children seemed positive about the interview experience. The children who expressed a continuing need for professional support already had ongoing contacts through school or health care. The research interviews for the study were conducted in accordance with the ethical guidelines for research in the human sciences and with the permission of the Regional Ethical Review Board in Uppsala, Sweden (Id. 2015/504).

## Results

The results are structured chronologically from narratives about life before to life after the support program, with meanings and changes highlighted. All the participants have been given fictitious names and any personal details that could reveal identity have been removed or altered in order to maintain confidentiality.

### Life Before the Grief Support Program

In the interviews, both children and parents were asked to recall life before their participation in the grief support program. Several children stated in a few words that they did not know anyone else who had lost a parent through suicide, and that they had avoided talking about their parent's suicide outside the family. Sometimes they said they did not feel the need to talk about it within the family either. Most children described how they had tried to live as before with a main strategy being to keep quiet about thoughts and emotions associated with parental loss. The children's more limited narratives were contextualized by the parents' descriptions. Most children were living with both parents at the time of the suicide, although a few parents had separated. In the latter cases, the child either shared accommodation, living every other week with each parent, or lived only with the remaining parent based on an awareness of the reduced caring capacity of the deceased parent due to psychological and/or substance-related problems. All the children in the study told how they had had a valued relationship with their deceased parent, and the parent's suicide had clearly caused a profound loss in their lives.

Most parents had been in contact with the children's schoolteachers to inform them of the parental suicide and the children's classmates were often also informed. Some children explicitly stated that they felt a sense of security knowing that their teachers and peers knew what they had been through, and some also reported that they had been offered professional support from a school counselor or nurse. Two children told of experiences of being bullied before their parent's suicide. In these cases, the information provided to the school seemed to have reinforced a sense of otherness when it did not lead to sympathy and support.

The children's narratives show that the prerequisites for mourning can vary. Many described an active social life on the outside, involving school, peers and spare time interests, but with grief vying for attention on the inside. Others described a situation dominated by grief and loneliness. Vanja, 12 years old, lacked friends and used to go into the school toilet to cry by herself. In retrospect, she reflected on the importance of the grief support camp: “I felt more alone before—that it was just us. Then when you came to the camp, it was like “it's not just us, there are many others as well'.”

#### Suicide as a Traumatic and Stigmatizing Event

In the parents' narratives, a situation of chaos, loneliness and actively seeking professional help dominated their descriptions of life before participation in the grief support program. Kristina is a case in point: “I was a single mom with two children living at home and one that had moved out, and I felt very alone. I started to search on the Internet and found this and felt in my stomach that ‘I need help’.” Petra depicted the abrupt change in their family life: “Of course it was a shock when it happened and Johanna found him and I wasn't at home and all… my parents moved up to us and stayed the whole summer actually, until school started.” A few parents, like Petra, described how they had received emotional and practical support from relatives and friends in their social networks, which was much appreciated in their vulnerable situation. More common, however, was for parents to speak about experiences of stigmatization, and lack of understanding and support. Lisa, the mother of a 6-year old boy, described how she stopped talking about her son's father in their social circle after encountering negative responses to his suicide, such as hurtful comments or avoidant behaviors. She reflected on the social judgements and insecurities surrounding suicide and remembered an incident in childhood, when her mother had talked about a mother who had died by suicide, that had affected her own understanding of suicide.

“She has destroyed her children's lives,” she said. And this mother became a monster in my eyes. It was so awful, you couldn't even touch the subject, that was the feeling I got. Zero sympathy or understanding for the mother, that she could have needed help, or that she maybe was suffering or.… No, it was just… she was demonized, and the children would get hell.

Similarly, Annika, the mother of a 14-year old boy, compared the social responses to suicide to those after more “normal” deaths, a difference that she believed hinders communication and support-seeking after suicide: “They don't know what to say….If you'd said that ‘he was killed in a car accident’ oh that would've been ‘so tragic’ and ‘incredibly sad’, but when someone *did it to himself* it's another story. That's why it's so hard to talk to someone who has not been through the same thing.” Kristina fell ill with a chronic illness after her husband's suicide. She described how she and her teenage children were left alone in this challenging situation.

We've become alone (deep breath). Now it may be that I've also been ill. That people withdraw for that reason too. So, I don't know if it's been double for us, but friends and acquaintances have just disappeared. You'd think that when something like this happens, relatives might show up to help out with the kids, to support the kids and such, but no.…

In the narrated material as a whole, the suicide stigma and related difficulties of communicating about the parental suicide were a shared experience among the participants. They were also said to affect family interactions. The older children in particular sought to normalize themselves by avoiding talking about the suicide. Petra described how she negotiated between her conviction that children need to talk about the death of their parent to process the loss, and her daughters' resistance to talking about their father's suicide: “I haven't had a hard time saying that Mats took his own life, but I've restrained myself for the children's sake, because they were not ready. I understand that, since there are so many taboos and such about it.”

#### The Decision to Participate

The parents described how they had found out about the BRIS grief support program mainly through local self-help organizations or social networks for suicide-bereaved adults on Facebook. Several children in the study said they were hesitant, or even protested, when their remaining parent had suggested they participate in the camp. Ivar, 14 years old, remembered that his mother had already made up her mind so there was no point in him protesting: “Well *then* I wasn't very into it (laughs). It felt like a really unnecessary and boring thing to do, but mom just went “this is great, let's go!”, and we kind of had no choice, we just had to go with her.” Other children were positive about going. Anders, 11 years old, for instance, told of his need to meet others and to talk about his parental loss experience: “It's hard to explain, but I thought it'd be fun to go there because you'd get to meet others and talk about *it*.” Later he added that he was bullied at school and had never shared this experience with a peer.

All the parents considered that it might be conducive to the grieving process to go away as a family and focus on their suicide loss experience, in addition to meeting other families in a similar situation. Descriptions of some children's reluctance to participate—especially among the teenagers—also appeared in the parents' narratives. Erika, the mother of two teenage daughters, said: “I thought primarily of the girls, that… yes that they'd get to meet other children who have also lost a parent… and exchange experiences and see that it's not just *them*.” She convinced her oldest daughter who was unwilling to go that it would be good for the family. Mona, the mother of two boys, found it helpful to be supported in a home visit by the BRIS leaders on how to respond to her teenage son's resistance.

It was good that the children got to meet some of them as they would meet later, and one of them talked to Ivar and said: “you're not so into this, are you?” (laughs). They said that “well, teenagers are usually a bit negative before, but they're the ones who are the most positive after” (laughs). Then it was easier for me to “force” him to come along.

However, the parents' experiences also differed. Petra took the initiative to participate after her oldest daughter expressed a desire to meet others in the same situation.

Johanna said that she wanted to meet others who'd experienced the *exact same* so…. Maybe it's difficult to meet someone who has been through the exact same, but here she could meet others who are in a very similar situation. So, then I made up my mind and realized somewhere that this is going to be tough and heavy, but I still wanted us to do it.

### The Perceived Meaningfulness of the Grief Support Program

The meanings that the children and parents attributed to their participation in the BRIS grief support program are outlined below. They perceived the encounters with other suicide-bereaved persons, which contributed to support exchange and normalization, to be the most meaningful, but also the help gained to construct destigmatizing understandings of suicide.

#### The Importance of Connection and Normalization

Many children and parents expressed relief at having had an opportunity to meet other suicide-bereaved. This was described as having a normalizing effect that counteracted the reported suicide stigma in their daily lives. The importance of connection and normalization was mainly stressed in the youngest children's tangible appreciation of and joy at having met other suicide-bereaved children and was more specific in the older children's and parents' narratives. Hugo, 7 years old, just wanted to contribute one thing to the interview. He sat down with his back straight and stated in loud and determined voice: “I think you should get to stay *longer*… and I'd like to come back. In 1 year, there are 12 months and each month I think you should get to go there for 1 week.” Agnes, 7 years old, exclaimed: “We got 1 day less than the others because Vanja (her sister) got chickenpox. 1 DAY LESS.” She summarized her experience: “I think it's good that there are more who have parents who've died, but it's not so good that they've died.” Similarly, 9-year old Mira said: “It's nice in a way that you feel that you're not alone.” The children described how they formed new relationships mainly through the playful activities that took place between the grief-oriented group exercises. However, the exercises and conversations in the group meetings represented the backdrop against which this community was created, through a silent awareness of their shared experience of parental suicide. The social parts of the camp, the playful activities such as table tennis, floorball, and crafts with their new-found friends in their spare time, were the main interest, while their narrations about the content of the group meetings were more limited. Anders described his own, and he presumed the other children's, focus of attention during the camp.

I think it was fun because you got to meet new people and I made new friends. Err that's it really. We kids probably didn't think much about *why* we were there—that it would help us—we didn't really think about that. When we were doing [the exercises] then we thought more, but there was also free time and then you thought of it more as a get together with friends.

When the group meetings were discussed, the children became serious and lowered their voices, which indicated that these were a sensitive subject, probably because they were closely connected to their parent's death—something which most children said they had used to avoid thinking and talking about in everyday life. Elvin, 11 years old, may have been representative of many of the children in terms of how he perceived the more grief-focused conversations: “Yes, it was quite fun, when we didn't talk about what… when we did crafts and stuff… and had juice and biscuits and so on and yes… but it was *really hard* when we talked about what had happened.” Not many of the children described what they took from the group exercises and conversations, but Vanja said that it was helpful for her to talk about her own grief experience and listen to others. She said that she recognized herself in another girl's telling but, while she listened, she became aware of the time difference in their loss experience; that is, having lost a parent recently compared to having managed for several years without the deceased parent.

It was good to get to see how others felt. I don't think there were so many who wanted to talk, but there were some who wanted to tell like *everything*. I recognized myself quite a lot and then there was a girl who said: “I forget my dad more and more and then it feels like I'm letting him down.” I feel the same… So, she has managed without her father for one year and I've managed without mine for 5 years—there's a *little* difference.

Vanja likened the grief support camp to a place where broken hearts could heal. She thought back to when they were crafting in her group: “I remember that I painted a broken heart. Then I took glue plus BRIS and glued the heart together. BRIS attracts broken hearts and glues them together.”

Kristina believed that it was good for her teenage son and daughter to meet other young people who were affected by a parent's suicide, with the explicit purpose of normalizing them in relation to suicide: “I think it helped a lot to see other *ordinary children*—that they weren't strange in any way. Because that's how you've felt…stared at, everyone was talking about us…and you felt very alone.” As she drew on her own experiences, she added that she too found comfort in the meetings with similar people mourning a suicide: “Yes spontaneously, as awful as it may sound, precisely that there are others in the same situation, similar boat, that there are more like *us*.” Erika was also grateful to have met other suicide-bereaved families: “I thought it was great to be there, both for me and for the girls, and to see that *we're not alone* in this and just talk to others who are in the same situation and share experiences.” Louise, the mother of two boys, stressed the significance of these encounters with reference to her 11-year old son's negative peer experiences. She could see that he was supported in his grief by an awareness that he was not alone in his situation, and his still ongoing relationships with other children from the camp. In fact, all the children said in their interviews that they were pleased to have participated. This account by Louise gives a good description of the development that could be seen among the children during the camp stay, and especially the teenagers who had initially expressed doubts about participating.

What I remember as the absolute best of all moments on both support weekends was to see these 13-year-olds who had been so quiet and introvert in the beginning. When you had heard their parents' stories about how they…everything they had said and done and enticed to get them there…and on the Sunday, after lunch when we were going home, they ran around and hugged each other and jumped for joy and hugged all the adults and “see you soon,” and were so happy. Yes, I get chills.

Finally, Lisa, who had previously described how she had been silenced in her social circle, summed up her experience:

The community, the warmth, the love and how you didn't feel alone, I took all of this with me. It was very important, how to relate to it all. For us, it feels natural to talk about it, not for everyone, but there you got a space to do it and meet other families. It was sad, but less lonely and isolating. You didn't feel strange or that you should apologize for what had happened… that you should be ashamed. Otherwise, I wouldn't have endured.

As the above shows, the participants were keen to express how much they had appreciated the opportunity to meet other suicide-bereaved families for normalization and support-exchange. In fact, these encounters stood out in the participants' narratives as the most meaningful contribution of the grief support program. However, when people come together based on an expected similarity, such as in the case of suicide bereavement, there is always a risk of disappointment and heightened exclusion if such a sense of belonging does not arise. One father described such a lack of connection. He explained this himself by saying that he is an introverted person who does not like to share emotionally charged topics. In his bereavement story he also positioned himself as different from the others; he said that most parents had struggled with their spouse's mental ill-health before suicide, while he did not consider that his wife was mentally ill. Another potentially negative aspect of this community building is the psychological burden of listening to others' detailed stories about traumatic deaths. One mother told how much she appreciated the group community, but at the same time found engaging with the others' suicide loss experiences emotionally draining.

#### Support to Construct Destigmatizing Understandings of Suicide

One educational element of the program that drew special attention in the interviews from both children and parents was how they had been assisted in age-appropriate ways to construct destigmatizing understandings of suicide. The notion that suicide is caused by a “thought disease,” depression or emotional suffering was introduced and discussed in the groups, adapted to the age of the children and the circumstances of death described. The program theme had been accentuated by research about the negative effects of suicide stigma on mourning families, of which many participants already had lived experience.

In the youngest age group, which was children aged between four and six, the leaders drew a large head on a whiteboard and painted thoughts and emotions in different colors to illustrate the variations in a healthy mind. Gradually, they painted this over in black to show how dark thoughts shaped by a thought disease dominated the mind. Finally, at the time of suicide, only a small light remained in the deceased parent's mind, which was all the love for the child. What the parent may have felt and thought before the suicide was discussed, as well as what the parent could have done instead of dying. The children became involved and told how they thought the parent felt sad and lonely, and had difficulties finding a solution; they concluded with the leaders that it was sad that the parent had not sought help. The youngest children did not recapitulate this meaning construction in their interviews, but several of the older ones did.

In the older age group, Johanna, 13 years old, described how the leaders had likened the depressed mind to a withering garden. The gardener can usually nurture most plants but some are impossible to revive. Eventually, as the illness progresses, the lush garden turns into a withered landscape, and the gardener/depressed individual has difficulties finding new solutions: “I thought it was good that we talked about suicide as a thought disease and that it was nobody's fault. It was that person's *thoughts*… it all came down to that.” Her reflection shows how this interpretation of suicide could help to counteract self-blame and stigmatization, since her conclusion opposes the notion that *someone* is to blame for suicide. Similarly, Vanja developed her thoughts on her father's suicide:

Yes, it was a thought disease. I don't know what it's called… [I: Depression?] Yes. That you only think sad thoughts…. It wasn't *he* who did it, it was the *thoughts*. He couldn't think of anything joyful in life. He just thought that life was wrong and everything.

Through their repeated interpretations of suicide in their interviews, both girls illustrated how they had internalized a destigmatized understanding of their fathers' suicides long after the intervention. Anders used the same knowledge in his meaning construction. He saw his father's suicide as the result of negative thoughts and self-loathing: “He had a thought disease. We heard that he died from a thought disease and when you've got a thought disease you believe that you're bad and can't manage anything. It's like ‘It would all be better without me’.” He told how he found this explanation reasonable and comforting. The suicide could even be understood as an act of love, since he stated that his father believed he was a burden to his family, and that suicide would thus relieve the family of suffering. These destigmatizing meaning constructions were also attributed meaning in the parents' interviews. Mona told how this explanation of suicide had been recurrently re-established in family conversations by her two sons.

That was something they could talk about. Then I thought that they [the leaders] must have talked about it in a good way, since they could talk about it (laughs). I think it's so important because it's where I think it's difficult. On the one hand, there are many taboos among the children, that you sit with “I wasn't worthy enough for dad” or something like that. I think this [new information] really came through. Because Elvin recounted it and it's so nice to hear it from him. He told me what he'd realized so he really understood. I think this was one of the most important things for the kids.

Most parents expressed gratitude for this help to find a shared meaning construction of suicide in grief; they described how it reduced feelings of guilt and shame, and became a model for how they could continue to talk with their children about suicide. One mother, however, held on to her resentment toward her former husband who she considered had failed in his parental responsibility to seek help instead of “deciding to leave.”

### Narratives of Change in the Grieving Process and Life

In their interviews, the children displayed insights about grief as a lifelong process and talked about how they used to cope with it in their daily lives. There was a general perception among the children that thoughts and emotions connected to their parental loss felt to various extents more manageable than before. Most parents, in turn, reflected on what they had learned and how they used this knowledge in family life. Their increased understanding of children's grief was explained as helpful and contributing to more supportive family communication. Many also told how the program had contributed a more hopeful view of life ahead. The main narrated changes are described below.

#### Children's Strengthened Agency and Management of Grief

In the children's talk about their lives now, they drew on lessons from the program and displayed agency by exemplifying how they had adjusted their coping strategies to grief-related emotions and needs. In the group sessions, the children had shared their loss experiences and strategies in discussions and were normalized and supported in their responses to loss. The children also processed their grief individually. In one exercise, the children had created their own first *aid kit*—a red glittery box in which they put written or drawn tips for themselves about what they could do to manage grief. Several children remembered the advice they had given themselves. Some brought out the saved boxes but declared that they no longer used them. Instead, they described the strategies they now used. Their primary advice to themselves from the program was to engage in different activities such as: “go outdoors and ride a bike,” “bake cookies,” “build with Lego” or “play with a friend,” aimed at distraction to avoid thinking about the deceased parent. These and similar distraction strategies were being used. Ivar is a case in point: “I don't know, I try *not* to think about it (laughs). I do something else like scroll on YouTube or something. Focusing on something else is good.” Selma, 9 years old, explained how she tried to activate herself to counteract painful thoughts, but on other occasions allowed herself to be sad.

Sometimes I just walk around the apartment and: “okay, what can I do?” Then I start watering the flowers or something… and I make drawings and put glitter on and stuff… I want to be alone. Or I go to bed and cry a little bit… then I fix with my mobile phone.

Johanna, described how she had also adjusted to recurring moments of mourning connected to her father's suicide: “I just think about it…and I know that it'll pass. Because I think about it every day and you probably will for the rest of your life.” She added: “If I'm really sad I talk to my friends or mum.” Anders described how his primary strategy was to talk to someone if he felt sad, although at that time he was not experiencing a need to do so. “I usually talk about it at home, but now I don't do that so much.” He had also received support from a school counselor. Agnes, 7 years old, said that she used to seek comfort by cuddling with her hamster, but she said: “Now I run to mom instead.” Finally, Vanja described her coping strategy in grief. “When I'm sad I listen to Sofia and Alio.” She had earlier explained that it had been helpful to listen to the other children's narrated experiences at the grief support camp and she had continued to listen to others' grief experiences in the form of song texts. In the interview, she played specific songs that she found had a healing effect on her. She reflected: “When I listen so Sofia, I feel like it's me who's singing. It's a beautiful song (she exhales). If something is worse though, like with her, you can really feel ‘what a good life I have’.”

In general, the children in the study showed an awareness of their emotions and needs in grief, and conveyed a perceived manageability in taking care of these. Grief was discussed as an ongoing process. Intrusive thoughts of the loss were said to come and go but were not considered dangerous or to be avoided at all costs. Instead, the children portrayed how they had created a space for grieving in their daily lives (27). In all the children's narratives, the remaining parent and sometimes other adults and friends were considered available resources that they could turn to for support.

#### Increased Parental Awareness of Children's Grief Responses and Needs

Most parents repeated pieces of advice that they had received from the group leaders and described them as helpful in interactions with their children. One main lesson that was raised was to strive for open and honest family communication about the suicide in order to support their children's meaning construction of their parent's suicide. At the enrollment interview, the parents were asked to tell the children that their parent had died by suicide, but not all of the children were aware of the detailed circumstances surrounding the death. Thus, the parents told how they had initiated conversations with their children after the camp to ensure that they received at least the basic information. Manuel sought advice regarding *when* and *how* he should tell his two preschool-aged daughters about their mother's suicide.

For me, it was very important to be able to reach a… new way of dealing with the big issue with the children. Because I didn't really know when to tell… I've always been so busy with… their lives and their primary needs and then came this question: “when should I tell them?” Should I tell them when they're 13 and ask: “dad *how* did mom die?” I didn't know. BRIS had a psychologist who explained why it's important for them to know the truth. For me, that was *the big thing*. Going through this was very important for me, and to get it done the right way. Talking openly with the children feels good and like you're doing the right thing.

Louise was also unsure about how much she should disclose to her 9-year old son about his father's suicide. She described how her son was affected by his participation and the encounters with other suicide-bereaved children in such a way that he had later asked for more information. With guidance, the mother was able to meet his needs.

Hugo told me that someone in his group had said that his father had shot himself…. Then he told me: “my dad died, and I don't know anything.” I remember talking to the leaders about it. I told them that “Hugo doesn't know” and I brought it up in the parent group, because I'd initially been advised by a child psychologist that when it comes to such small children one shouldn't tell them about the event. So, I thought I'd handled it correctly but then I realized that it was a huge mistake that he didn't know. It also emerged in conversations we had afterwards that he'd been thinking about horrible, bloody things out in the garage… and that wasn't at all what had happened….

Among other things, the parents were informed that children who lose a parent through suicide may experience feelings of anger, shame and blame, which may be difficult to articulate in grief. Mona had been inspired to help her sons express such complicated dimensions of grief. Despite her efforts, however, her sons did not show much interest in talking about their emotions. She then drew on other advice from the program and changed her position.

It was a frustration I had that they didn't talk. I needed to pull it out of them. We talked about Jakob in positive terms like “do you remember…?” and such, but not about….But then Lena (one of the leaders) said “you may think that it's a monolog, but it's a dialogue that goes on in the children's heads. You just can't hear it. They'll think on it, but it may not be you who gets to take part in it.” Then I felt that's so true. Because if you've got something to say you should say it, even though you don't get a response. They listen and then it continues…and if they feel a need to talk about it, they'll do just that.

#### Open Communication Within the Family and Social Network

As noted above, the parents became aware through the program of how they could support their children in an open and honest family communication. The program content promoted such communication and the parents also frequently referred to specific exercises to illustrate the changes they had noticed. For example, each family created a collage by cutting out pictures from magazines to portray who the deceased parent was. Once complete, the children and parent presented the deceased parent to the other participants and received positive confirmations. The exercise was intended to help reconnect to the parent as a person separate from the suicide. Lisa described how it motivated her and her preschool-aged son to remember and talk about his deceased father: “The collage was such a good activity; to do it together, but also that the children could explain and present. It became such a good thing, to be reminded of his father, because it felt like we didn't talk enough about him.”

Annika reported the changes she saw in her teenaged son in terms of him opening up to her in grief. He was an only child and had not talked about his father's suicide at all before their participation in the program.

Our lives have really been affected by these weekends. They've been absolutely crucial. They made such a *difference*. Just knowing that there are more. Because it's a huge difference to participate here than going to a regular crisis group. It's not the same *at all*. Lars doesn't talk much, but after this he opened up and he has others to talk to as well.

Sibling relationships were sometimes also said to have improved. Kristina noticed how her teenage son and daughter began to share their grief after their participation and went to their father's grave together. Even communication in the families' social networks was commonly mentioned to have been enriched by the lessons from the grief support program. Louise described how her oldest son had spoken to a few friends about his father's suicide before his participation, which set a rumor in motion. After the program, he started to set boundaries for *when* and with *whom* he wanted to talk about his father's suicide, while her younger son, who had never told anyone that his father died by suicide, started telling the other children and teachers at his preschool and placed a photo of his father on his cloakroom shelf. Overall, participation in the grief support program was said to have contributed to a process of destigmatization, which made both parents and children feel more comfortable about talking about their parental loss in their social networks and less sensitive about the responses of others.

#### A Changed View of Life Ahead

The children who participated in the study often expressed a positive commitment to leisure activities and friends, and seemed preoccupied with life here and now, while the parents more often reflected on the family's future and expressed a more positive view of life ahead. In addition, the children who had reported previous experiences of bullying and loneliness in grief told of an improved situation after the program through new friendships and increased manageability of grief. Louise exemplified how participation in the grief support program could be described as a turning point in the participants' lives. She described how the more playful family activities had helped her to reconnect with her former self as an active and playful mother, before her husband's suicide 2 years before.

I thought that the family activities were really great, because I didn't have the strength…I managed quite well to take care of everyday life here at home, but I wasn't….If I think back on myself from *that* time, I don't know if I *ever* laughed, that's how it was. I've always been an inventive person who likes to go outdoors and do things, but it disappeared quite a bit because I had no energy and no desire or anything…all my energy went on just surviving every day. So I was so incredibly grateful to be in a context where someone else organized the activities, where we got to laugh together again, and to do fun things that everyone enjoyed.

She explained how she had been revitalized through the meetings and the positive change she experienced in their family interaction. All in all, this was said to have helped her create a better life for herself and her children.

Going was a turning point in my life. After that I could *live* again. It's so clear to me that I also began to relate to the children in a more natural way again, as it should be, not in a catastrophic way. Not in worry and such…but that we can trust that maybe we can also get to live and have a good time. Although this horrible thing has happened, we can probably actually do just that.

## Discussion

### Discussion of the Main Results

This narrative evaluation has showcased the significance suicide-bereaved children and parents attributed to their participation in a family-based grief support program. The program is arguably similar to a “compassionate communities approach”[c.f. (32)] given that it aims to educate and support suicide-bereaved families to facilitate their coping with loss as a complement to existing healthcare. In addition, it draws attention to their situation and needs to a general public. First and foremost, both children and parents valued the opportunity to meet other families affected by a parent's suicide; this was said to contribute support exchange and normalization in relation to suicide as a stigmatizing death. An urgent need among suicide-bereaved family members to meet similar grievers has been reported and discussed in several studies, in order to share their experiences, and to learn from others, for example, how to manage the pain and the transition between life before suicide to life after suicide, [e.g., (16, 33–35)]. The community that emerged for most of the participants in this study was based on an overall quest to regain meaning and joy in life after suicide, and this was supported through the program structure and its content.

The result of this study backs up evidence from previous studies (19, 20) that a structure that offers a variation between “grief work” and outdoor recreation or play is particularly appropriate for children, because it supports relationship building and fits with children's developmental need to “go in and out of grief” to avoid suffering overly intense emotions (27, 29). This has also proved suitable for suicide-bereaved families as a whole. The parallel themed sessions in children and parent groups, as well as the family-oriented grief exercises, were said to facilitate a continuing dialogue in the family about sensitive issues related to the parent's suicide [cf. (5, 6)]. Similarly, the playful activities strengthened family interactions and supported a reorientation from the heavy yoke of grief to cheerful escapades in the family. The latter were said to contribute the hope of emotional survival of the suicide and for brighter prospects. This is an important finding, given how bereaved families can lock themselves into grief and tend to do fewer activities together after a parent's suicide (10). The overall empowering social context of the grief support camp stands in stark contrast to the descriptions of the social barriers to support in the participants' daily lives linked to a prevailing suicide stigma. Like so many people mourning a suicide in the family, the narratives in this study echo how both suicide-bereaved children and their parents usually struggled alone before arriving at the grief support camp.

A central element of the program is the help to construct a tolerable meaning of parental suicide that does not stigmatize the bereaved family. Through the meaning reconstruction in the program, the participants learned that the parent suffered from a psychological condition influenced by destructive thoughts, and ultimately sought to escape emotional pain, which clarified that *no person* was to blame. The children in particular voiced release from self-blame and their self-esteem appeared restored through this explanatory model and the specific message that they were not unloved or rejected by the deceased parent (8). From a social constructionist and narrative perspective on loss, grief and trauma (36), such meaning reconstruction in the wake of loss is desirable. It addresses the *crisis of meaning* (11) that arises when suicide challenges previously taken-for-granted beliefs about this life world and the self. It also has the potential to combat stigma and contribute to reconciliation in relationships, including with the deceased, restored identities and even post-traumatic growth (36, 37). The above meaning reconstruction has health benefits too, since feelings of blameworthiness have been associated with grief difficulties, complicated grief, PTSD, depression and other mental health difficulties (38), while the role of self-forgiveness in suicide bereavement has been linked to a decrease in depression and suicidality among suicide loss survivors (39). In addition, the results show that even very young children, can benefit from being included in family communication on and meaning reconstruction of the parent's suicide (40). The open and honest communication that the program encourages between parents and children opposes a more protectionist stance toward children and empowers their position in grief. This is in line with current recommendations that children should preferably be informed of the true circumstances of a death in a developmentally adapted manner (8, 35, 41).

The parents highly valued the educational elements of the program on childhood bereavement after parental suicide and gave several examples of how this knowledge was implemented in family life. In general, the parents expressed increased confidence about their capacity to support their children, which was confirmed in the children's reports on the parent as a resource in their grief. Altogether, this supports the assumption, based on research, that when parents are supported in grief and in their parenting, this has positive effects on children [see (18)].

The narrated changes in the children's grief processes and lives indicated an increased sense of coherence (28). In their telling of experience, the children seemed empowered in their relation to the parental suicide and in their dealing with grief. They appeared to have integrated non-stigmatizing comprehensions of their parent's suicide and performed agency and manageability in grief by recounting their purposefully used coping strategies. They also demonstrated interest in relationships and social activities they found meaningful. This adds to the research on the effects of a family-based approach to work with suicide bereaved children [cf. (5, 6)].

Finally, this study describes an ethical approach to research interviews with children on sensitive subjects. The children's responses showed that even though emotions related to a parent's problems and death can surface in an interview situation, they appreciated being able to contribute their experiences to research in the way they chose.

### Limitations

A prerequisite for children's participation in a grief support camp of this kind is the remaining parent's ability to identify such a need. A challenge for research and practice is thus to reach the suicide-bereaved children who do not have a supportive remaining parent—the children who themselves have several risk factors for developing ill-health and suicidality. This study is biased in this regard since all the parents reached out for professional help. In addition, the self-selected sample of participants constituted about half of all the families who participated in the BRIS grief support program. It can be assumed that those who were particularly positive about their participation wanted to “give back” out of gratitude or to help gain the program permanent status. However, those with experiences of a different kind might also be motivated to air their opinions in order to improve program content or prevent such a process gaining legitimacy. In telephone contacts with parents who refrained from participating, their decision was motivated by an overly pressing life situation as a single parent and/or problems with their children's functioning and well-being linked to grief, for which they had sought professional help. All, however, expressed gratitude for their participation in the program. A further limitation of the study was that the children were not invited individually by telephone, but only through an age-adapted and personally addressed letter. The parents were subsequently asked whether they and/or their children wanted to participate. Based on their decision, plans were made for a home visit or the contact was ended. It is possible that more children, especially teenagers, would have been more inclined to participate in the study if they had been in direct contact with the author. Lastly, the long-term follow-up in the study made it possible for the participants to reflect on how their lives had been affected by their participation in the grief support camp 18 months later, but this design made it difficult for the youngest children to remember. From a child perspective, a longitudinal approach with an initial short-term follow-up and further follow-up would have been more appropriate.

## Conclusion

This family-based grief support program in the format of a weekend camp with a particular focus on children's grief has been shown to have helped to open up family communication and strengthen family resources for coping with a parent's suicide. It has great potential to counteract complications in suicide-bereavement—not least those induced by stigmatizing attitudes and self-imposed blame for suicide—and to promote health and well-being in this vulnerable group. Such a psychoeducational measure is thus considered to be a highly effective intervention with a relatively low cost compared to other traditional approaches. However, although influenced by social relationships and norms, grief is a unique and highly personal process, which means that not everyone will benefit from professionally led support groups. For example, individual preferences can make it difficult to disclose one's own experiences or listen to the stories of others. Therefore, other professional measures should also be considered with the aim of meeting the often-overlooked needs for support after a suicide in the family.

## Data Availability Statement

The raw data supporting the conclusions of this article will be made available by the authors, without undue reservation.

## Ethics Statement

The studies involving human participants were reviewed and approved by Regional Ethical Review Board in Uppsala, Sweden (Id. 2015/504). Written informed consent to participate in this study was provided by all the children aged 8 and above, and all the children's legal guardians.

## Author Contributions

The author confirms being the sole contributor of this work and has approved it for publication.

## Funding

The study was funded by FORTE: Swedish research council for health, working life and welfare (Grants 2018/01052).

## Conflict of Interest

The author declares that the research was conducted in the absence of any commercial or financial relationships that could be construed as a potential conflict of interest.

## Publisher's Note

All claims expressed in this article are solely those of the authors and do not necessarily represent those of their affiliated organizations, or those of the publisher, the editors and the reviewers. Any product that may be evaluated in this article, or claim that may be made by its manufacturer, is not guaranteed or endorsed by the publisher.

## Footnotes

^1^In this article, “children” refers not only to biological age, but also to young people's position in relation to the deceased parent. The child participants in the study were both younger children and teenagers.
