# Supplementary material for: A Narrative Evaluation of a Grief Support Camp for Families Affected by a Parent's Suicide
Source: Front Psychiatry. 2021 Nov 30;12:783066. doi: 10.3389/fpsyt.2021.783066 (PMC8686190; doi:10.3389/fpsyt.2021.783066)
Supplement: Supplementary file 1 [file Presentation_1.pdf]

Grief support program

First camp stay

Day 1

Families arrive and are installed in their rooms.

Shared lunch between families and leaders.

Welcome meeting and Contextualization: All families have lost a parent through suicide. Short presentation on leaders and family members, and the parent who they are there to remember. Schedule review and practical information.

Group session: *"Creating the group"*. Discussions/exercises to get to know each other. Arranging a memory table in the room with photos of the deceased parents.

Shared dinner.

Outdoor quiz walk with flashlights. One leader accompanies each family.

Leisure time with games, crafts, table tennis and floorball.

Day 2

Shared breakfast.

Gathering with all the families. Presentation of the activities of the day.

Group session: *"Why does someone die by suicide?"* Information about suicide and discussion. *"The family then and now: what happened?"* The children draw their families and inform the others about the parent who died.

Shared lunch.

Group session: *"What has changed?"* Children and parents fill in a "film strip" covering three squares of what life was like before the suicide and when it had just happened, and now in their current situation. Group discussion about changes in person, relationships, school/work, and so on.

Family exercise: *"Remembering the deceased parent"*, creating a collage. One leader supports each family.

Outdoor activities in mixed teams. Stations with different exercises and scores.

Shared dinner.

Music quiz in mixed teams.

Leisure time with games, crafts, table tennis and floorball.

Day 3

Shared breakfast.

Group session: *"Grief responses and emotions"*. Information about suicide bereavement and coping. The children create an "emotion box" illustrating how they perceive themselves on the outside and inside. Emotions are identified, normalized, and discussed in relation to how they are managed in everyday life.

Summary of the three days with all the families. Next support weekend is in four months.

Shared lunch before departure.

Second camp stay

Day 1

The families arrive and are installed in their rooms.

Shared lunch between families and leaders.

Welcome meeting. Presentation round to reconnect. Schedule review and practical information.

Group session: *"Recreating the group"*. Update: The participants inform each other about their current situation and how it feels to be back. *"Grief over time"*: Information about the grieving process. Exchange of grief and coping experiences in retrospect – what has changed over time? *"My grief/others' grief"*: Individual exercise and group discussion. The children fill in a circle about how much each family member grieves and reflect on their own space for grieving. *"Questions I wanted to ask but have not dared"*: Children and parents write questions to each other between the groups.

Playful activities: Musical chairs, and balloon and name games. The children make face masks and the parents must guess who their child is.

Leisure time with games, crafts, table tennis and floorball.

Day 2

Shared breakfast.

Gathering with all the families. Presentation of the activities of the day.

Group session: The parents answer the questions from the children and vice versa. *"Who was my mother/father?"*: The children receive a memory book with preprinted questions about their parent to answer. Group discussions about memories and grief. The parents learn about children's grief.

Outdoor activities: Hide and seek and family challenges.

Shared lunch

Group session: *"How do I take care of myself"*. Discussions on the emotions identified in the earlier exercise: bleeding heart, super angry, jealous, and so on. The children write tips to themselves and put them in a first aid kit. They receive answers to their questions from their parents, and vice versa. The children are encouraged to ask their parents new questions in the future.

Family exercise: The families create a "memory bottle" with colored layers that represent different memories of the deceased parent.

Shared dinner.

Treasure hunt for the children, who needs help from their parents.

Leisure time with games, crafts, table tennis and floorball.

Day 3

Shared breakfast.

Gathering with all the families.

Group session: *"What is my future?"* A fourth square representing the future is added to the "film strip" the participants filled out during their first camp stay. Reflections on changes since last time. Participants draw or write what they hope for the future.

Family exercise: Each family member writes letters to themselves on how they want the future to look, beginning with “I want…”, “I wish…” and “I will…”. The letters are to be opened at a certain time in the future.

Summary and program ending with all the families: Each child and parent group shows something that they have talked about and created during their group sessions.
